# Meconium Proteins Involved in Iron Metabolism

**DOI:** 10.3390/ijms25136948

**Published:** 2024-06-25

**Authors:** Ewa Skarżyńska, Artur Jakimiuk, Tadeusz Issat, Barbara Lisowska-Myjak

**Affiliations:** 1 Department of Laboratory Medicine, Medical University of Warsaw, 02-097 Warsaw, Poland; 2 Department of Obstetrics, Women’s Diseases and Gynecologic Oncology, National Medical Institute of the Ministry of the Interior and Administration, 02-507 Warsaw, Poland; jakimiuk@yahoo.com; 3 Center for Reproductive Health, Institute of Mother and Child, 01-211 Warsaw, Poland; 4 Department of Obstetrics and Gynecology, Institute of Mother and Child, 01-211 Warsaw, Poland; tadeusz.issat@imid.med.pl; 5 Department of Biochemistry and Pharmacogenomics, Medical University of Warsaw, 02-097 Warsaw, Poland

**Keywords:** meconium, fetus, haptoglobin, ferritin, ceruloplasmin, transferrin, NGAL, lactoferrin, myeloperoxidase, calprotectin

## Abstract

The lack of specific biological materials and biomarkers limits our knowledge of the mechanisms underlying intrauterine regulation of iron supply to the fetus. Determining the meconium content of proteins commonly used in the laboratory to assess the transport, storage, and distribution of iron in the body may elucidate their roles in fetal development. Ferritin, transferrin, haptoglobin, ceruloplasmin, lactoferrin, myeloperoxidase (MPO), neutrophil gelatinase-associated lipocalin (NGAL), and calprotectin were determined by ELISA in meconium samples obtained from 122 neonates. There were strong correlations between the meconium concentrations of haptoglobin, transferrin, and NGAL (*p* < 0.05). Meconium concentrations of ferritin were several-fold higher than the concentrations of the other proteins, with the exception of calprotectin whose concentration was approximately three-fold higher than that of ferritin. Meconium ceruloplasmin concentration significantly correlated with the concentrations of MPO, NGAL, lactoferrin, and calprotectin. Correlations between the meconium concentrations of haptoglobin, transferrin, and NGAL may reflect their collaborative involvement in the storage and transport of iron in the intrauterine environment in line with their recognized biological properties. High meconium concentrations of ferritin may provide information about the demand for iron and its utilization by the fetus. The associations between ceruloplasmin and neutrophil proteins may indicate the involvement of ceruloplasmin in the regulation of neutrophil activity in the intrauterine environment.

## 1. Introduction

Although the importance of iron for a healthy pregnancy is well recognized, how iron transfer to the fetus is regulated during pregnancy is not well understood [1,2]. During the fetal and neonatal periods, both overload and deficiency of iron may be responsible for dysfunction of the developing organs [1,3,4].

In humans, iron incorporated into proteins controls formation of reactive oxygen species, thus protecting cells from oxidative stress [1,2,3,4]. Mammalian iron metabolism is tightly regulated at both cellular and systemic levels [5]. The complex regulation of systemic iron homeostasis is dependent upon tight coordination of absorption, storage, and transport of iron in the body, which involves the participation of proteins which perform these various biological functions [4,6].

Apart from the early stage of iron transport from the maternal circulation through the placenta, little information is available concerning the transfer of iron after it is taken up by the syncytiotrophoblast and its further distribution to the fetal organs as demanded [1,7].

Meconium is a biological material specific to fetal development, having characteristic consistency and color. The components of meconium accumulate in the fetal bowel starting at 13 weeks of gestation and are excreted after birth [8,9]. Meconium, as confirmed by proteomic studies, includes numerous proteins, with varied biological properties and functions in immediate contact with the developing fetus [10]. The selection of proteins functionally related to the regulation of iron metabolism and the assessment of their interdependencies in the meconium of the newborn presented in this paper can determine a panel related by common biological tasks for maintaining homeostasis in the intrauterine environment.

In the present study, meconium was used to determine the concentrations and their correlations of several proteins which employ different regulatory mechanisms to efficiently conserve and recycle iron in the body. The selection of proteins determined in meconium and listed below was based on their well-established role for assessing systemic iron homeostasis and on the recognized clinical significance of their serum measurements.

**Ferritin (FER)**: An intracellular iron storage protein, stores surplus iron after all the cellular needs are fulfilled and releases it in the face of an acute demand. An indirect marker of iron status and a highly specific and sensitive parameter for diagnosis of iron deficiency [5,11].

**Ceruloplasmin (CP)**: A multicopper oxidase and antioxidant, capable of oxidizing Fe^2+^ (ferrous iron) into Fe^3+^ (ferric iron), facilitating the incorporation of Fe^3+^ into transferrin (TRF), an acute-phase protein, forms complexes with lactoferrin (LF) and myeloperoxidase (MPO) during inflammation [12,13].

**Transferrin (TRF)**: The major serum iron-binding protein, an essential biochemical marker of body iron status, responsible for ferric-ion delivery, transports iron through the blood to various tissues, acts as a balance between reticuloendothelial iron release and in bone marrow uptake [11].

**Haptoglobin (HP)**: A plasma protein with the highest binding affinity for hemoglobin, may have a crucial role in heme-iron recovery, an important modulator of iron homeostasis [14].

Neutrophil granule proteins were another group of proteins determined in meconium. Their well-established properties indicate their likely involvement in the regulation of iron metabolism in the intrauterine environment [15,16,17].

**Lactoferrin (LF)**: A non-hemic iron-binding protein found in the secondary granules of neutrophils, with a high Fe^3+^–binding capacity, the major functional protein in maintaining human health due to its antioxidant, antibacterial, antiviral, and anti-inflammatory activities [18,19].

**Myeloperoxidase (MPO)**: A lysosomal enzyme mainly found in the azurophilic granules of most activated neutrophils, produces highly oxidizing substances from hydrogen peroxide and halogens, a potential trigger to vascular injury, linked to both inflammation and oxidative stress [20,21].

**Neutrophil gelatinase-associated lipocalin (NGAL)**: An acute-phase protein, found in the secondary granules of neutrophils and secreted after their activation, involved in iron delivery to and export from cells via a transferrin-independent mechanism. NGAL binds siderophores, plays a role in regulating iron activity, modulates iron transport as part of antibacterial immunity, and is an inflammatory mediator [22,23].

**Calprotectin (CAL)**: A heterodimeric complex of two non-covalently associated calcium-binding proteins, S100A8 and S100A9, is stored in large amounts in the granulocyte cytosol (40–60% of cytosolic protein content), and has both intracellular and extracellular functions. Calprotectin is involved in inflammatory processes and/or inhibition of microbial growth, chelates iron, and deprives bacteria of this essential nutrient [24,25,26].

The aim of the study was to assess the homeostasis of iron metabolism in intrauterine fetal development by determining the concentrations and interdependencies in neonatal meconium proteins commonly recognized in laboratory diagnostics for the assessment of iron status.

## 2. Results

Table 1 presents the concentrations of serum-derived and neutrophil-derived proteins measured in parallel in 122 meconium samples collected from 122 neonates.

The concentrations of serum-derived and neutrophil-derived proteins are expressed as µg/g meconium. Calprotectin and ferritin were found at the highest concentrations. The highest levels of dispersion of the concentration measurements (a coefficient of variation greater than 100%) were demonstrated for transferrin, haptoglobin, lactoferrin, and myeloperoxidase.

The ratios presented in Table 2 indicate the differences in the proportions of serum-derived proteins in meconium and the proportions of their concentrations in serum. The assessment of these differences is based on the concentration ranges for individual proteins sourced from www.urmc.rochester.edu Encyclopedia (accessed on 15 April 2024). The mean value [mg/dl] was calculated by adding the lowest and the highest value in the range and dividing the result by two (ferritin 0.016, transferrin 298.0, haptoglobin 120.0, ceruloplasmin 27.5).

The ratios of ferritin to transferrin, haptoglobin, and ceruloplasmin were approximately 5000–500,000-fold higher for meconium than for serum. The ratios of haptoglobin to transferrin and ceruloplasmin were approximately 10-fold lower for meconium than for serum.

Table 3 compares the ratios of the meconium concentrations of neutrophil-derived proteins to their concentrations in serum. The mean serum concentrations of neutrophil-derived proteins [ng/mL] are based on the literature (lactoferrin 2420.0 [27,28], MPO 75.0 [27,29], NGAL 123.0 [30,31], calprotectin 1259.0 [24,32]. Serum concentrations of these neutrophil proteins were not included in the www.urmc.rochester.edu › Encyclopedia (Accessed on 15 April 2024). To obtain the mean values of the concentration of these proteins in the serum, the available information from the PubMed database was used.

The ratios of calprotectin to lactoferrin, MPO, and NGAL were approximately 10-fold higher for meconium than for serum. The ratios of lactoferrin to MPO, NGAL, and calprotectin were approximately two-fold lower for meconium than for serum.

**Figure 1:**
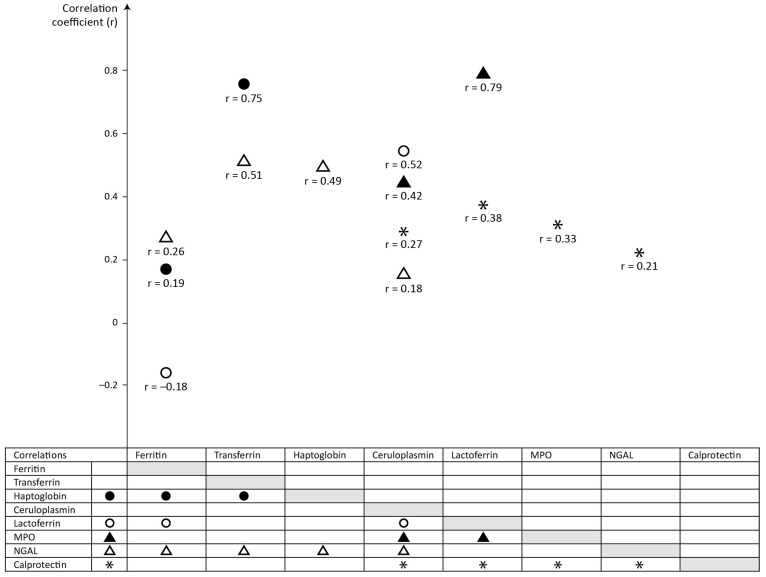
Correlations between the meconium concentrations of proteins.

Table 4 and Figure 1 show statistically significant correlations between serum-derived and neutrophil-derived proteins in meconium.

The values of coefficients of correlation presented in Table 4 and Figure 1 demonstrate the strongest association (*r* > 0.7) between meconium haptoglobin vs. transferrin and lactoferrin vs. MPO. No significant associations were found between ceruloplasmin- and serum-derived proteins (ferritin, transferrin, haptoglobin), but there were significant correlations between ceruloplasmin- and neutrophil-derived proteins (lactoferrin, MPO, NGAL, calprotectin). Meconium NGAL concentrations significantly correlated with the concentrations of serum-derived proteins.

## 3. Discussion

Based on the results of this study, the measurements in meconium of a panel of proteins with a well-established use as serum tests of iron status are proposed as a new perspective from which to search for new biomarkers of iron homeostasis in the fetus. It has been recognized that the complex mechanisms underlying systemic iron homeostasis involve specific proteins with various biological functions [5,11,12,13,14,15,16], but little is known about the role(s) individual proteins play to control iron metabolism in the intrauterine environment [3]. High coefficient variation and large ranges between concentration measurements demonstrated for these proteins in the meconium may inform us about the possibility of their active involvement in the regulation of intrauterine processes related to the development of the fetal organism. According to Georgieff [3], fetal iron can be divided into three compartments: red cells, storage iron mostly in the liver, and non-heme non-storage tissue iron. At term 70–80% of fetal iron is present in red blood cells as hemoglobin, 10% in tissues as myoglobin and cytochromes and the remaining 10–15% stored in reticuloendothelial and parenchymal tissues as ferritin and hemosiderin [7]. Iron endowment of the fetus is entirely dependent on iron transport through placenta from the maternal circulation, involving iron transporters and regulation of placental expression of these proteins, and iron recycled from aged cells [1,2,3,14]. The lack of biomarkers to be determined in fetus-specific biological materials is a limitation to our understanding of the distribution of iron from maternal circulation, intrauterine recycling of iron, its transport to fetal organs for storage, and the adequate supply required for fetal needs.

The present study used a panel of proteins whose individual biological properties are utilized in routine laboratory serum tests to assess iron metabolism in the body. Protein concentrations were measured in the meconium, which is easily collectable from a newborn [8]. As far as we know, the determinations of proteins involved in the regulation of iron homeostasis presented in this publication are carried out for the first time in newborn meconium and have no comparative results so far. An attempt to compare the mutual proportions of proteins determined in the meconium with the theoretical knowledge in the literature about the proportions of the same proteins in serum was intended to bring closer the knowledge about the homeostasis of iron metabolism in these two clinical materials representing separate body spaces. The ranges of protein serum concentrations were sourced from the literature [24,27,28,29,30,31,32] and the website www.urmc.rochester.edu › Encyclopedia. Significant differences were found between the ratios of haptoglobin and ferritin concentrations to the other proteins in the meconium and serum.

In humans, iron is incorporated into proteins as a component of heme in hemoglobin, myoglobin, cytochrome proteins, myeloperoxidase, and nitric oxide synthetase [4]. The ratios of haptoglobin to transferrin and ceruloplasmin in the meconium which were approximately 10-fold lower than those calculated for serum may indicate that haptoglobin concentrations tend to decrease in the meconium. This observation may be accounted for by haptoglobin depletion, as it forms a complex with heme iron transported to the liver [14]. High correlations of haptoglobin with transferrin and NGAL suggest that in the intrauterine environment other transport proteins may significantly assist in the recycling of iron from senescent and damaged red blood cells. Taking into consideration various mechanisms of iron transport and associations within the triad consisting of haptoglobin, transferrin, and NGAL found in the meconium, these proteins may serve as a diagnostic panel for the assessment of intrauterine homeostasis of iron both recycled and maternal.

Ferritin plays essential roles in cellular and systemic iron homeostasis and sequesters excess intracellular iron, thus protecting cells from oxidative stress and storing it for future use in conditions of deficiency or high demand [3,5,33]. In clinical practice, low ferritin is highly specific as an indirect marker of deficiency of total body iron stores. High ferritin may indicate increased iron stores, but it may also be a result of ferritin release from damaged cells, its increased synthesis and/or increased cellular secretion triggered by a variety of stimuli such as cytokines, oxidants, hypoxia, oncogenes, and growth factors [34].

According to the literature, ferritin and other proteins determined in the meconium in this study share a tightly regulated role in the uptake and storage of toxic iron [5,6]. On the other hand, the lack of strong correlations of meconium ferritin with the other proteins involved in the regulation of iron homeostasis may suggest that ferritin plays an independent biological role in the intrauterine environment.

Unlike in adults, where most of the luminal iron taken into the intestinal mucosal cells is stored within the cells as ferritin and subsequently lost in the feces when the cells exfoliate at the end of their lifespan [35], in a fetus a direct contact with the immature intestinal barrier of meconium containing ferritin at high concentrations might lead to increased penetration of toxic iron. Cellular and systemic ferritin levels are not only crucial indicators of iron status but are also important markers of inflammation and of immunological and malignant disorders [1,2,7]. Fetal liver can control the availability and distribution of iron to other anatomical sites, which is evident from the experimentally confirmed effect of reduced iron stores in fetal hepatocytes on the support of hematopoiesis by supplying iron [33]. There is growing evidence reported in the literature indicating that iron dysregulation determined by the delicate balance between cellular iron usage and surplus, leading to toxicity from excess quantities of free reactive iron, is responsible for the pathogenesis of various metabolic, neurological, and inflammatory diseases [6].

A question arises concerning the level of the iron saturation of ferritin in meconium and whether it may actually compromise fetal development. High ferritin concentrations found in the meconium may be the aftermath of a completed delivery of iron to the fetal organs, and variations in the meconium ferritin levels are likely to result from the dynamics of this process. Kämmerer et al. show the role of nurse macrophages in the systemic iron transport to erythroid progenitors, although it is unclear which transport proteins, transferrin, ferritin and heme, are involved [33].

The present study found a 10-fold increase in the ratios of calprotectin concentration, which represents approximately 45% of the total cytosol protein in neutrophiles, to the concentrations of lactoferrin, myeloperoxidase, and NGAL calculated for meconium compared to serum. Neutrophils are one of a host of innate immune cells detected at the maternal–fetal interface, but little is known about the role they play in the intrauterine environment. Decreased concentrations of neutrophil granule proteins compared to calprotectin concentrations may be evidence of the anti-inflammatory immune milieu with effects on fetal growth, characteristic of the third trimester of gestation [15,16].

Additionally, the findings of this study suggest that ceruloplasmin may be individually involved in the regulation of neutrophil activity in the meconium. This observation confirms an experimental study by Sokolov et al. [17], who found that ceruloplasmin can form complexes with lactoferrin, which is an iron-binding protein, and with heme-containing myeloperoxidase and can be regarded as an anti-inflammatory factor.

Interestingly, the correlations between NGAL and the other iron transport proteins such as transferrin and haptoglobin were high. NGAL as an extracellular iron carrier by binding to siderophores, small iron-binding compounds typically secreted by microorganisms, interacts with receptors on the cell surface, transporting iron into the cell and releasing it inside. The characteristic properties of NGAL suggest that it may be involved in local iron control. On the other hand, NGAL, a newly described adipocytokine, acts as an inflammatory factor [4,23]. A study by Yin [23] confirmed the individual role of NGAL in fetal development.

The limitations of this study are the lack of total iron determination in the meconium and information about the iron saturation of proteins, including ferritin as the main storage protein, as well as transferrin, lactoferrin, and NGAL involved in intracellular and extracellular of iron transport. The characteristic relationships between proteins regulating iron metabolism in newborn meconium raise a number of questions about the possibility of their application in clinical practice. Further research should clarify the biological significance of high ferritin concentration in meconium and the role of this parameter in the assessment of the iron endowment of the fetus. The interconnected panel of the proteins transferrin, haptoglobin, and NGAL, independent of the variability of ferritin concentration, suggests the possibility of its use to understand intrauterine iron transport. The relationships of neutrophil proteins with other proteins regulating iron homeostasis in meconium may provide new diagnostic tools for controlling the role of these cells in the intrauterine environment. Measurements of the study proteins in cord blood and maternal blood would increase understanding of iron metabolism in meconium.

## 4. Materials and Methods

### 4.1. Study Group

Included in the study were a total of 122 newborn infants of 36–41 weeks of fetal age with birth weight above 2500 g, born by cesarean section (*n* = 33) and by vaginal (*n* = 89) in the Department of Obstetrics, Women’s Diseases and Gynecologic Oncology at the Central Clinical Hospital of the Ministry of the Interior and Administration (present name The National Medical Institute of the Ministry of the Interior and Administration) in Warsaw. The recruitment process lasted 18 months (2021–2023). The inclusion criteria were singleton deliveries with clear amniotic fluid, and the newborns did not pass meconium in the uterus or during delivery, but after birth. The women giving birth had no anemia or clinical signs associated with an acute or chronic hypoxic event. The exclusion criteria were multiple pregnancies, anemia of pregnancy despite the treatment, coloured amniotic fluid, and weight of the newborn after delivery below 2500 g. Written informed consent was obtained from the parents or guardians prior to inclusion of the infants in the study.

This study was performed in line with principles of the Declaration of Helsinki and approved by the Local Committee for Human Experiments at the Central Clinical Hospital of the Ministry of the Interior and Administration (The National Medical Institute of the Ministry of the Interior and Administration) in Warsaw (Decision No. 71/2011).

### 4.2. Meconium Collection and Homogenate Preparation

A sample of the first meconium was collected from a neonate in the hospital ward from the nappy with a disposable spatula and transferred into a 50-mL graduated plastic tube and frozen at −20 °C for up to 7 days, and next, a meconium homogenate was prepared. The empty tubes were weighed prior to adding the meconium and reweighed after filling. The date, time, and weight of each meconium collection were recorded. The weight of the meconium sample (*n* = 122) ranged from 0.484 to 9.899 g (mean ± SD 5.105 ± 2.442; median 5.174).

To prepare the homogenate, PBS (Phosphate Buffered Saline pH 7.4) was added to a tube with the meconium: one part by weight of meconium per four parts by weight of PBS added in two equal portions. After the first PBS portion was added, the tube was placed on a hematology mixer for 15 min and then shaken in a horizontal position using an LP300Hk shaker for 1 h. After adding the second PBS portion, the procedure was repeated. The homogenate was transferred to Eppendorf tubes and stored at −80 °C. Prior to protein measurements, the homogenates were thawed for 24 h in a refrigerator. Next, they were mixed on a hematology mixer at room temperature for one hour.

### 4.3. Laboratory Methods

The meconium proteins were measured by ELISA using Assaypro LLC ELISA kits (St. Charles, MO 63301, USA, www.assaypro.com) (accessed on 15 April 2024) according to the manufacturer’s instruction for each individual protein. The following kits were used to determine the concentrations of neutrophil proteins in the meconium: AssayMax^TM^ Human Lactoferrin ELISA Kit, AssayMax^TM^ Human Lipocalin-2 ELISA Kit, AssayMax^TM^ Human Myeloperoxidase (MPO) ELISA Kit, AssayMax^TM^ Human Calprotectin ELISA Kit. The following ELISA tests were used to determine the concentration of proteins of serum origin in the meconium: AssayMax^TM^ Human Ceruloplasmin ELISA Kit, AssayMax^TM^ Human Haptoglobin ELISA Kit, AssayMax^TM^ Human Ferritin ELISA Kit, AssayMax^TM^ Human Transferrin ELISA Kit.

Measurements were performed in accordance with the principles of Good Laboratory Practice (GLP) in duplicate, and then the mean value was calculated and reported as a final concentration.

### 4.4. Statistical Analysis

Statistical analysis using Statistica Version 13 (StatSoft Inc., TIBCO Software Inc., Palo Alto, CA, USA) was performed. In the first stage, the normality was assessed for each protein and the ratios were calculated by the Shapiro–Wilk test. The measurements are presented as mean ± SD (standard deviation); median; min-max; CV (coefficient of variation, %) for the proteins and as median and rank for the calculated ratios. Protein ratios were calculated for each individual meconium sample. The Spearman’s correlation coefficient (r) was calculated for assessing correlations between the concentrations of individual proteins in meconium. The statistical significance level was assumed as *p* < 0.05.

## 5. Conclusions

Measuring in meconium, a biological material specific to the newborn, of the concentrations of proteins commonly used as serum tests of iron status could be a new approach to assessing iron homeostasis in the environment of the developing fetus. The high coefficients of correlation calculated for the meconium concentrations of haptoglobin, lactoferrin, and NGAL demonstrate that they work together to maintain the metabolism and transport of iron in the intrauterine environment. The meconium concentrations of ferritin which were several-fold higher than the concentrations of the other proteins involved in iron metabolism indicate the individual role of ferritin in the accumulation and distribution of iron during fetal development. The associations of ceruloplasmin with neutrophil granule proteins suggest its potential as a biomarker for neutrophil activity in the intrauterine environment.

## Figures and Tables

**Table 1:** Concentrations of serum and neutrophil concentrations in meconium, n = 122 neonates/samples.

Protein (µg/g)	Mean ± SD	Median	Range	CV (%)
**Serum-derived Proteins in Meconium**				
Ferritin	78.6 ± 49.6	66.2	13.0–286.3	63.1
Transferrin	55.9 ± 109.1	12.6	1.1–655.7	194.9
Haptoglobin	1.2 ± 1.4	0.7	0.56–12.6	111.9
Ceruloplasmin	38.7 ± 38.4	24.7	0.9–218.5	99.4
**Neutrophil-derived Proteins in Meconium**				
Lactoferrin	34.1 ± 61.4	13.4	0.9–322.1	179.8
Myeloperoxidase	2.3 ± 3.6	1.4	0.04–22.0	154.6
Neutrophil gelatinase-associated lipocalin	2.5 ± 2.3	1.7	0.6–13.6	89.6
Calprotectin	239.6 ± 203.4	196.7	12.4–1284.7	84.9

CV = coefficient of variation

**Table 2:** Ratios of serum-derived proteins in meconium to serum-derived proteins in serum.

Ratios	Meconium		Serum
	Median	Range	
Ferritin to transferrin	4.79	0.05–150.31	5.4 × 10^−5^
Ferritin to haptoglobin	72.65	5.03–394.51	1.3 × 10^−4^
Ferritin to ceruloplasmin	2.46	0.15–133.58	5.8 × 10^−4^
Transferrin to ferritin	0.21	0.01–20.53	18,625.0
Transferrin to haptoglobin	17.81	1.53–222.31	2.5
Transferrin to ceruloplasmin	0.62	0.01–91.20	10.8
Haptoglobin to ferritin	0.014	0.003–0.20	7500.0
Haptoglobin to transferrin	0.056	0.005–0.65	0.4
Haptoglobin to ceruloplasmin	0.035	0.003–1.18	4.4
Ceruloplasmin to ferritin	0.41	0.01–6.63	1718.8
Ceruloplasmin to transferrin	1.61	0.01–86.38	0.1
Ceruloplasmin to haptoglobin	28.35	0.85–313.14	0.2

**Table 3:** Ratios of neutrophil-derived proteins in meconium to neutrophil-derived proteins in serum.

Ratios	Meconium		Serum
	Median	Range	
Lactoferrin to MPO	12.65	0.90–111.93	32.27
Lactoferrin to NGAL	7.77	0.38–134.28	19.67
Lactoferrin to calprotectin	0.09	0.002–1.24	1.92
MPO to lactoferrin	0.08	0.01–1.11	0.03
MPO to NGAL	0.69	0.02–8.74	0.61
MPO to calprotectin	0.01	0.0002–0.10	0.06
NGAL to lactoferrin	0.13	0.01–2.66	0.05
NGAL to MPO	1.45	0.11–51.15	1.64
NGAL to calprotectin	0.01	0.002–0.64	01.0
Calprotectin to lactoferrin	11.75	0.81–559.52	0.52
Calprotectin to MPO	142.56	10.42–4559.29	16.79
Calprotectin to NGAL	93.88	1.55–604.32	10.24

MPO = myeloperoxidase; NGAL = neutrophil gelatinase-associated lipocalin

**Table 4:** Correlations between serum-derived and neutrophil-derived proteins in meconium.

Meconium Protein	Significant Correlations (*p* < 0.05)	
	Serum-Derived Proteins	Neutrophil-Derived Proteins
Ferritin	*vs* haptoglobin, *r* = 0.19, *p* = 0.038	*vs* lactoferrin, *r* = −0.18, *p* = 0.042 *vs* NGAL, *r* = 0.26, *p* = 0.004
Transferrin	*vs* haptoglobin, *r* = 0.75, *p* < 0.001	*vs* NGAL, *r* = 0.51, *p* < 0.001
Haptoglobin	*vs* ferritin, *r* = 0.19, *p* = 0.038 *vs* transferrin, *r* = 0.75, *p* < 0.001	*vs* NGAL, *r* = 0.49, *p* < 0.001
Ceruloplasmin		*vs* MPO, *r* = 0.42, *p* < 0.001 *vs* NGAL, *r* = 0.18, *p* = 0.047 *vs* lactoferrin, *r* = 0.52, *p* < 0.001 *vs* calprotectin, *r* = 0.27, *p* = 0.003
Lactoferrin	*vs* ferritin, *r* = −0.18, *p* = 0.042 *vs* ceruloplasmin, *r* = 0.52, *p* < 0.001	*vs* MPO, *r* = 0.79, *p* < 0.001 *vs* calprotectin, *r* = 0.38, *p* < 0.001
MPO	*vs* ceruloplasmin, *r* = 0.42, *p* < 0.001	*vs* lactoferrin, *r* = 0.79, *p* < 0.001 *vs* calprotectin, *r* = 0.33, *p* < 0.001
NGAL	*vs* ferritin, *r* = 0.26, *p* = 0.004 *vs* transferrin, *r* = 0.51, *p* < 0.001 *vs* haptoglobin, *r* = 0.49, *p* < 0.001 *vs* ceruloplasmin, *r* = 0.18, *p* = 0.047	*vs* calprotectin, *r* = 0.21, *p* = 0.020
Calprotectin	*vs* ceruloplasmin, *r* = 0.27, *p* = 0.003	*vs* lactoferrin, *r* = 0.38, *p* < 0.001 *vs* MPO, *r* = 0.33, *p* < 0.001 *vs* NGAL, *r* = 0.21, *p* = 0.020

MPO = myeloperoxidase; NGAL = neutrophil gelatinase-associated lipocalin

## Data Availability

The data presented in this study are available on request from the corresponding authors. The data are not publicly available due to privacy.
